# Investigation of Flexural Bearing Behavior of Corroded RC Strengthened with U-Type TRC

**DOI:** 10.3390/ma17051154

**Published:** 2024-03-01

**Authors:** Wei Xie, Jie Sheng, Zongjian Yu, Jiong Zhu, Binbin Zhou, Ke Chen

**Affiliations:** 1 School of Civil Engineering, Xuzhou University of Technology, Xuzhou 221018, China; xiewei@xzit.edu.cn (W.X.); zhujiong@xzit.edu.cn (J.Z.); 20211105138@xzit.edu.cn (K.C.); 2 Yangzhou Zhongkuang New Building Material Technology Co., Ltd., Yangzhou 225000, China; 3 School of Mechanics and Civil Engineering, China University of Mining and Technology, Xuzhou 221116, China; zongjian_yu@cumt.edu.cn; 4 School of Urban Construction, Changzhou University, Changzhou 213164, China; 00003487@cczu.edu.cn

**Keywords:** textile-reinforced concrete, RC beam, corrosion, flexural behavior, U-type strengthening

## Abstract

In this study, the flexural bearing behavior of corroded reinforced concrete (RC) beams reinforced with U-type Textile Reinforced Concrete (TRC) was investigated using a four-point bending loading method. Nine test beams were produced: one original beam, three RC beams with corrosion alone, and five corroded beams strengthened with U-type TRC. The analysis focuses on assessing the impacts of the steel corrosion degree and the number of textile layers on various aspects of the bending behavior, such as failure modes, bearing capacity, and load displacement curves, in U-type TRC-strengthened corroded beams. The experimental results revealed three distinct failure modes in the U-type TRC-strengthened corroded beams. TRC effectively enhanced the bearing capacity. With sufficient textile layers, it can be restored to the level of the original RC beams. Moreover, in the cases of severe corrosion in RC beams, the bearing capacity increased more significantly. The TRC also enhanced the ductility. Finally, a calculation equation for the ultimate bearing capacity of U-type TRC-strengthened corroded beams was presented and validated, demonstrating consistent alignment with the experimental data.

## 1. Introduction

Since their inception, reinforced concrete (RC) structures have been extensively utilized across various engineering domains, including buildings, roads, bridges, tunnels, and water conservancy projects. They are fundamental engineering materials. However, RC structures are susceptible to durability issues during extended utilization. Steel-bar corrosion is the most prevalent and severe concern of durability problems in RC structures [1,2,3]. The hazards associated with steel-bar corrosion encompass several critical aspects. (1) The decreases in bond strength between the concrete and steel and the decline in the effective cross-sectional area of the steel bars lead to a decrease in the load-bearing capacity of the structural components. (2) The rust volume expansion exceeds that of the original steel (often reaching two–six times its initial size) [4,5], resulting in concrete cover cracking and a subsequent reduction in the component’s stiffness and integrity. (3) The decreased ductility and alterations in failure modes collectively affect safety, applicability, and RC structural durability.

Before the 1990s, China constructed numerous RC bridges in the coastal regions to foster economic development. These bridges have experienced severe steel corrosion issues after a decade or two of service [6]. Among these bridges, a considerable portion exhibits a reduced bearing capacity, posing a substantial threat to road safety. The prospect of demolishing and reconstructing these bridges presents several drawbacks, including the generation of waste that is difficult to recycle and exacerbation of the existing tension between economic development and resource, energy, and environmental conservation [7]. In comparison, reinforcing bridges that exhibit early signs of corrosion is a more rational alternative.

Fiber-reinforced polymer (FRP) materials have been employed for enhancing corroded RC structures, owing to their notable advantages, including lightweight composition, ease of installation, high tensile strength, and robust resistance against corrosion. Research findings have indicated that FRP can effectively redistribute the tensile stress in corroded steel bars, improving the load-bearing capacity of deteriorated structures. Concurrently, adding an FRP layer, particularly when augmented with U-shaped hoop restraints at its extremities, significantly promotes the stiffness of the structure by effectively restraining deteriorated concrete [8,9,10,11]. Nevertheless, the prevalent utilization of organic materials, such as epoxy resin, as bonding agents in FRP systems presents certain limitations. These shortcomings include inadequate high-temperature resistance, suboptimal compatibility with concrete substrates, and challenges associated with their application on damp structural surfaces [12,13,14,15].

Recently, textile-reinforced concrete (TRC) has emerged as a promising alternative to FRP. TRC consists of one or more layers of textiles embedded in a high-performance cementitious mortar. These textiles may be composed of carbon, glass, basalt, or polypropylene benzobisoxazole (PBO) fibers. TRC has outstanding properties, including high tensile strength, lightweight composition, and resistance to corrosion. TRC also exhibits good durability. For example, the study of Verre and Sheng showed that FRCM (fiber-reinforced cementitious matrix is another name of TRC) exhibited excellent resistance to alkaline, hot water, and freeze-thawing environments [16,17]. Moreover, TRC is compatible with existing concrete and masonry structures, rendering it straightforward to install [18,19,20,21]. Franzoni et al. [22] performed direct shear tests on FRCM-masonry joints after capillary water absorption and salt crystallization cycles. The results show that FRCM-masonry joints exhibited excellency due to the permeable matrix that did not hinder the migration of the saline solution. Numerous studies have investigated the mechanical properties of RC structures reinforced with TRC, demonstrating its feasibility in enhancing the performance of RC structures. For instance, Yin et al. [23,24,25] conducted an extensive series of studies on TRC reinforcement for RC beams and columns, revealing that TRC can effectively augment the ultimate bearing capacity of these elements. Notably, this enhancement increased as the number of textile layers increased. Furthermore, TRC significantly improved the distribution pattern of bending cracks in the specimens, characterized by their fine and dense nature. Schladitz et al. [26] identified a remarkable rise in the load-bearing capacity of RC slabs that were enhanced with one and four layers of FRCM, recognized as an alternative to TRC. Specifically, the load-bearing capacity improved by 67% with one layer and 245% with four layers of FRCM. Additionally, the application of a four-layer FRCM system led to a notable reduction in slab deflection.

However, limited research has focused on the flexural behavior of TRC used to strengthen corroded RC beams. Bressan et al. [27] and El Maddawy et al. [28] explored the flexural behavior of FRCM strengthening in corrosion-damaged RC beams. Their findings indicated a significant enhancement in both the yield and ultimate capacity of corrosion-damaged specimens following FRCM strengthening. Specifically, the applications of C-FRCM (Carbon-FRCM) and PBO-FRCM successfully restored and even surpassed the ultimate load-bearing capacity of the non-corroded control specimens. The failure mode was ductile. Similarly, Elghazy et al. [29,30,31] conducted research on the flexural behavior of corroded RC beams strengthened with FRCM, adopting a single-sided reinforcement method. The experimental results demonstrated that FRCM could enhance the yield strength of corroded beams by 6% to 22% and the ultimate strength by 5% to 52%. Notably, despite the anchoring measures implemented at both ends of the reinforcement area, there were instances of FRCM delamination during loading, hindering the full realization of its potential.

To further investigate the flexural bearing performance of TRC in enhancing corroded RC beams and explore methods to avoid TRC delamination during loading, this study employs a continuous U-type TRC for the reinforcement of such beams. In this study, nine test beams were proposed: one reference beam that remained free from corrosion and reinforcement, three beams subjected to corrosion alone, and five beams reinforced with TRC following corrosion. The parameters encompassed the corrosion degree and number of textile layers. Comprehensive analyses were conducted to evaluate the ductility performance, load–displacement curves, bearing capacity, and failure modes. An equation for quantifying the flexural bearing capacity of a U-type TRC in reinforcing corroded RC beams was established.

## 2. Materials and Methods

### 2.1. RC Beam Design and Fabrication

**Figure 1:**
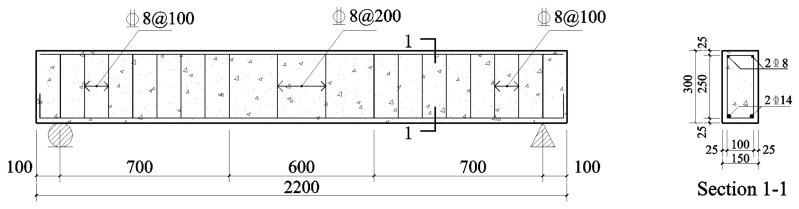
Reinforcement and dimensions of the original RC beam (unit: mm) [32].

The dimensions of the test beam included a length of 2200 mm, a width of 150 mm, a height of 300 mm, and a clear span of 2000 mm. In the tension zone, two HRB400 steel bars measuring 14 mm in diameter were arranged, and two HRB400 grade steel bars measuring 8 mm in diameter were positioned in the compression zone. For the stirrups, HRB400 steel bars with an 8 mm diameter were employed, with a pure bending section spacing of 200 mm and a shear span spacing of 100 mm. The concrete cover had a thickness of 25 mm. Nine test beams were fabricated: one original RC beam, three RC beams exposed to corrosion alone, and the remaining five beams reinforced with TRC following corrosion. The geometric dimensions and reinforcement configurations of RC beams are illustrated in Figure 1.

### 2.2. RC Beams’ Accelerated Corrosion Method

**Figure 2:**
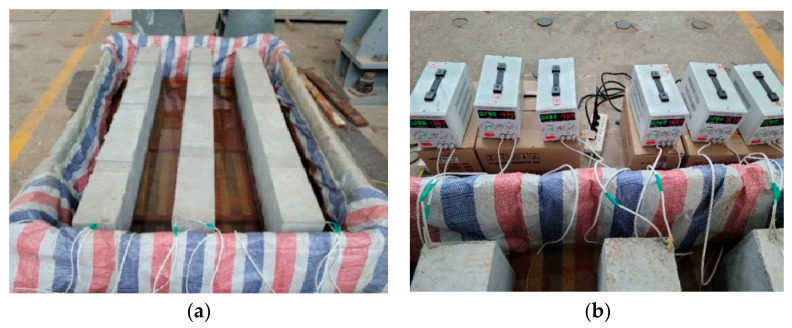
Illustration of the accelerated corrosion process: (**a**) wooden tanks accommodated with RC beams; (**b**) DC power.

Considering the time cost and experimental control, this study employed the electrochemical accelerated corrosion method to prepare the corroded specimens. The procedure involved the following steps. Before binding the steel bars, the insulation tape was wrapped around the outer side of all stirrups to mitigate the corrosion effects. After the RC beam was poured and allowed to cure for 28 d, it was placed in a specially designed wooden water tank (Figure 2a). A 5% NaCl solution was introduced, and the liquid level was meticulously regulated to ensure direct contact with the lower surface of the bottom steel bar of the beam. This was executed to induce as much uneven corrosion as possible. To facilitate the complete penetration of chloride ions into the concrete cover, the beam was immersed in the solution for 72 h before being subjected to electrification. Subsequently, a direct current was applied to the longitudinal reinforcement of the beam, maintaining a controlled current density of 300 μA/mm^2^ (Figure 2b). Throughout the electrification process, the current passing through each steel bar was monitored daily, and any rust products formed on the cathode were immediately cleaned to ensure corrosion efficiency. The degree of steel corrosion was quantified by the weight loss rate, which was calculated using Equation (1). The theoretical mass loss was determined using Faraday’s law, as expressed in Equation (2). This study targeted weight loss rates of 5%, 10%, and 15%, representing mild, moderate, and severe corrosion, respectively.

To determine the true corrosion degree of the steel bars, a 100 mm segment of the corroded steel bar was carefully cut following the completion of the bending test. Subsequently, a solution of 12% dilute hydrochloric acid was applied to remove the rust from the corroded steel bar. Subsequently, the corroded steel bar was thoroughly rinsed with distilled water and dried using a dryer. Finally, an electronic scale was utilized for weighing the corroded steel bars that were dried. The true corrosion degree can be computed by Equation (1):

(1) $$\eta =\frac{\Delta m}{m}$$

(2) $$\Delta m=\frac{MI}{Fnt}$$

where *m* denotes the quality of the uncorroded steel bar, *η* represents the corrosion degree, Δ*m* represents the quality of the steel bar after removing the rust, *F* denotes the Faraday constant, *N* denotes the chemical valence of the reaction electrode (2 in this equation), and *M* is the molar mass of iron.

### 2.3. Materials

a. Concrete

Commercial concrete was employed in the RC beams. Three concrete cubes, each with a 150 mm side length, were produced and subjected to a compressive test after the 28-day standard curing, according to the *Standard for Testing and Evaluating Concrete Strength (GB/T 50107-2010)* [33]. The resulting measured compressive strength was shown in Table 1.

b. Rebar

According to *Tensile testing of metallic materials-Part 1: Room temperature test method (GB/T228.1-2010)* [34], the measured mechanical properties of the steel bar are shown in Table 2.

c. Textile

**Figure 3:**
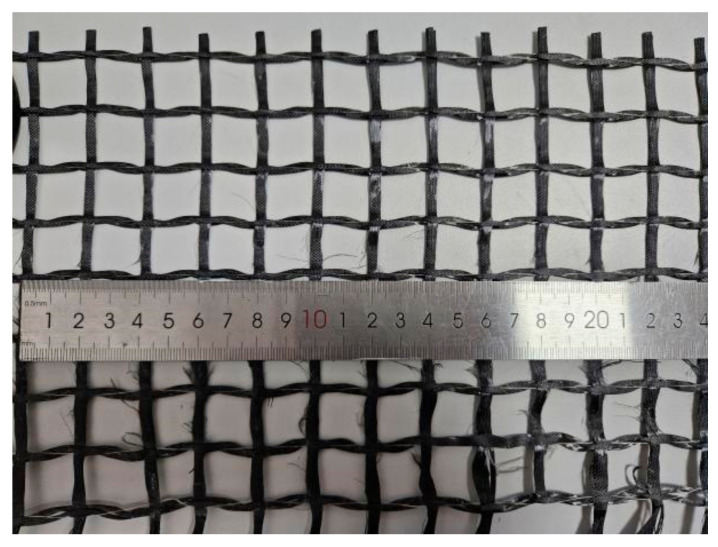
Carbon textile.

The textile employed in this experiment was a bidirectional carbon fiber woven mesh, measuring 20 mm × 20 mm (Figure 3). The specifications of the manufacturer for the mechanical performance indicators are presented in Table 3.

d. Matrix

In this experiment, fine-grained concrete served as the matrix material for the TRC system, and the proportions of the specific mix can be found in ref [35]. The cement used was ordinary Portland cement of type 52.5R, and the fly ash was first-class grade. A high-performance polycarboxylate water-reducing agent was selected as the water-reducing agent. Fine sand and coarse sand, with particle sizes of 40–70 mesh and 20–40 mesh, respectively, consist of ordinary quartz sand. To obtain the compressive strength of the fine-grained concrete, three cubes measuring 70.7 mm on each side were subjected to testing after 28 d of standard curing, following the guidelines outlined in JGJ/T70-2009 [36]. The results are shown in Table 4.

### 2.4. Strengthening Method

**Figure 4:**
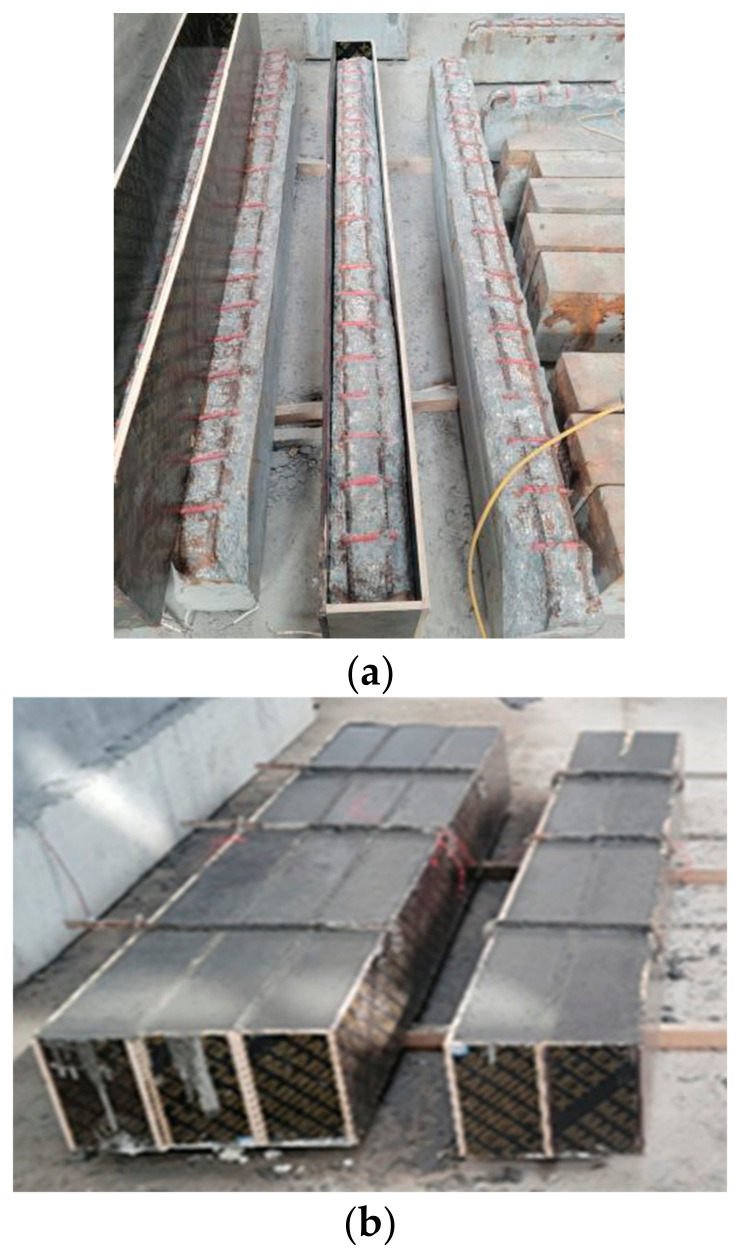
Restored and corroded RC beams: (**a**) eliminating the deteriorated concrete; (**b**) forming fine-grained concrete [32].

Following the completion of the accelerated corrosion, the concrete in the lower portion of the beam, approximately 50 mm in height, was removed using an electric pick to expose the steel bars. Subsequently, all rust products were meticulously polished. The formwork was then arranged around the beam, and fine-grained concrete was employed to restore the original cross-sectional dimensions of the specimens (Figure 4a,b). Subsequently, TRC was applied to reinforce these beams.

**Figure 5:**
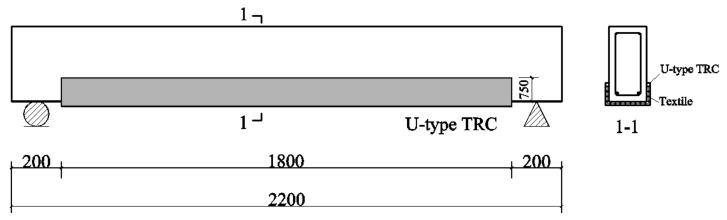
Replaced concrete cover and TRC-strengthening schemes (unit: mm).

The TRC layout followed a U-type configuration, which positioned the TRC on both the bottom and sides of the beam (Figure 5). The height of the TRC side arrangement was 75 mm, and the TRC on the bottom spanned a length of 1800 mm. Each specimen was designated according to the format M-A-LB, where A signifies the corrosion degree of the specimen, and LB indicates the number of textile layers employed. For example, M-5-L1 denotes a specimen with a corrosion degree of 5% and one layer of textile reinforcement.

### 2.5. Measurement Point Layout and Loading Plan

**Figure 6:**
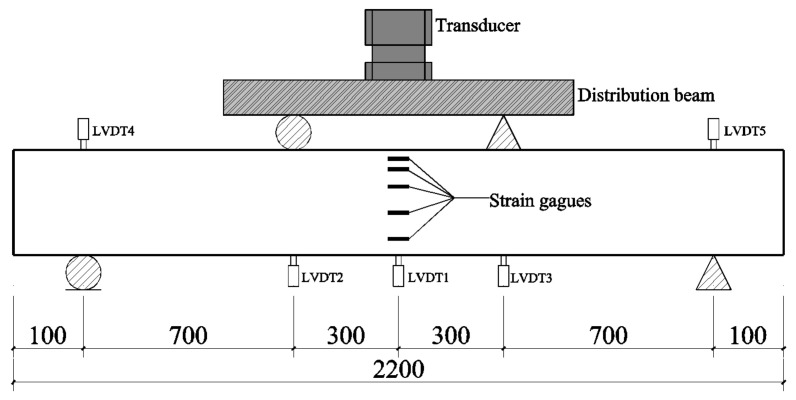
Measuring and loading point layout diagram (unit: mm) [32].

The measurement points and loading arrangements are shown in Figure 6. A four-point bending loading method was employed with symmetrically positioned loading points along the test beam, with a pure bending section spanning 600 mm. A 50-ton hydraulic jack was used for loading. Before the formal loading procedure, an initial load of 2.0 kN was applied and maintained for 5 min to eliminate any gaps between the test beam and its support. Subsequently, gradual unloading was performed before proceeding with formal loading. Formal loading was conducted in a stepwise manner, with increments of 5 kN at each level. Following each loading, a 10–15 min holding period was observed. Throughout this process, a crack observation instrument was utilized for measuring the crack widths, featuring a measurement range of 0.01–2 mm and an estimated reading accuracy of 0.01 mm. Strain gauges were employed with a length of 100 m situated at different distances from the top surface to measure the concrete strain, specifically at 0, 25, 50, 100, 150, and 225 mm from the top to the bottom of the beam. The tensile steel bar strain was monitored using 3 mm long resistance strain gauges. Additionally, displacement measurements were obtained at the support, loading point, and mid-span using a YHD-50 displacement meter, equipped with a 50 mm range and 0.1 mm accuracy. Data, including displacement, strain, load, and other relevant parameters at each measurement point of the test beam, were systematically collected using data collection equipment.

## 3. Results

### 3.1. Failure Mode

**Figure 7:**
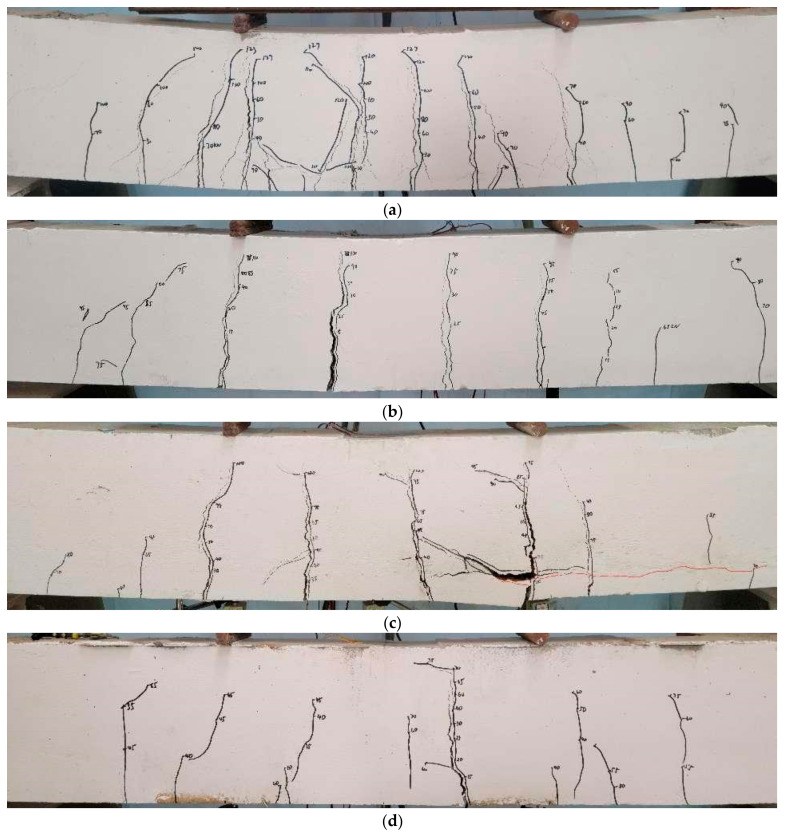

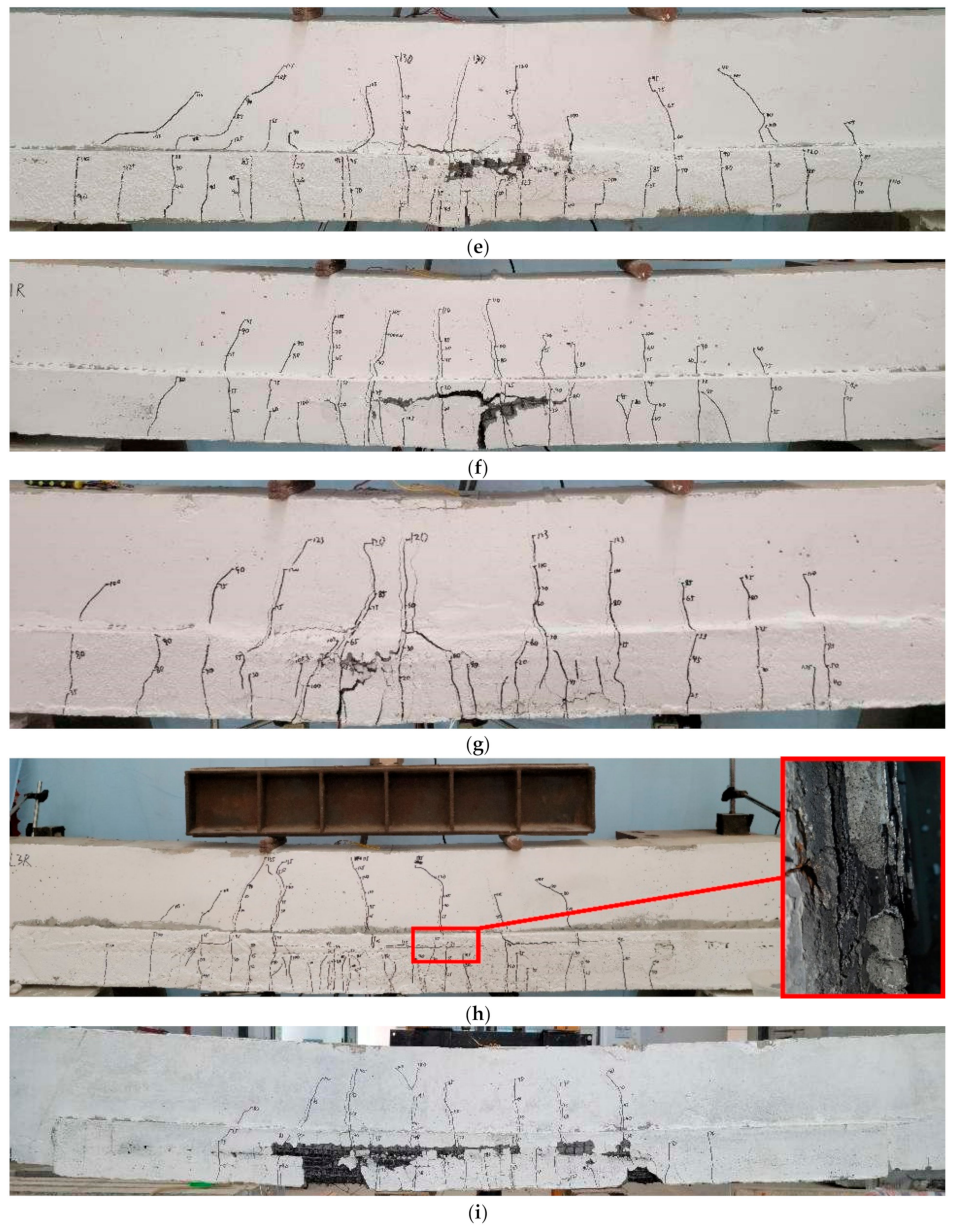
Failure modes of specimens: (**a**) M-0; (**b**) M-5; (**c**) M-10; (**d**) M-15; (**e**) M-5-L2; (**f**) M-10-L1; (**g**) M-10-L2; (**h**) M-10-L3; (**i**) M-15-L2.

The ultimate failure modes of each specimen are shown in Figure 7. In the case of the corrosion-only beams (M-5, M-10, M-15) and original beam (M-0), a bending failure mode was observed, where the tensile steel bars yield initially, followed by subsequent concrete crushing within the compression zone. The failure characteristics of corroded RC beams, where TRC was strengthened, were summarized as follows: the tensile steel bars yielded first, followed by failure of the textile layer, and ultimately, concrete crushing within the compression zone. Nevertheless, TRC exhibited varying failure modes categorized as follows:

The first type of failure resembled the bending failure observed in RC beams. This mode of failure was evident in three beams, namely, M-5-L2, M-10-L1, and M-10-L2. As the load intensified following the yielding of steel bars, there was an audible indication of certain fiber bundles undergoing tension, indicating an uneven stress distribution within the fiber bundles. As the load increased further, the rest of the fiber bundles were unable to resist external forces and underwent sudden separation. Owing to the internal force redistribution, an abrupt surge in the compressive stress emerged within the concrete located in the compression zone, causing its crushing.

The second type of damage involved the delamination between the textile and its matrix. This destructive process commenced with obtaining the tensile steel bars, followed by the debonding of the textile from its matrix. Consequently, the reinforced beam was unable to sustain the load, with the compression zone concrete being crushed. In the case of the damaged strengthened beam, only a portion of the fiber bundles was pulled apart. Specifically, this damage occurred in specimens M-10-L3 due to the proximity of the textile.

The third type of damage involved a fiber bundle slip, coupled with matrix detachment. Specimen M-15-L2 exhibited this type of damage. After reaching the yield point, the beam experienced slippage between the fiber bundles and the matrix. With the continued increase in load, matrix detachment became more pronounced, followed by a loss of load-bearing capacity. This occurrence was attributed to the relatively short curing time of the TRC layer in this beam, causing a weaker bonding performance between the matrix and textile, in contrast to the other beams.

### 3.2. Bearing Capacity Analysis

**Figure 8:**
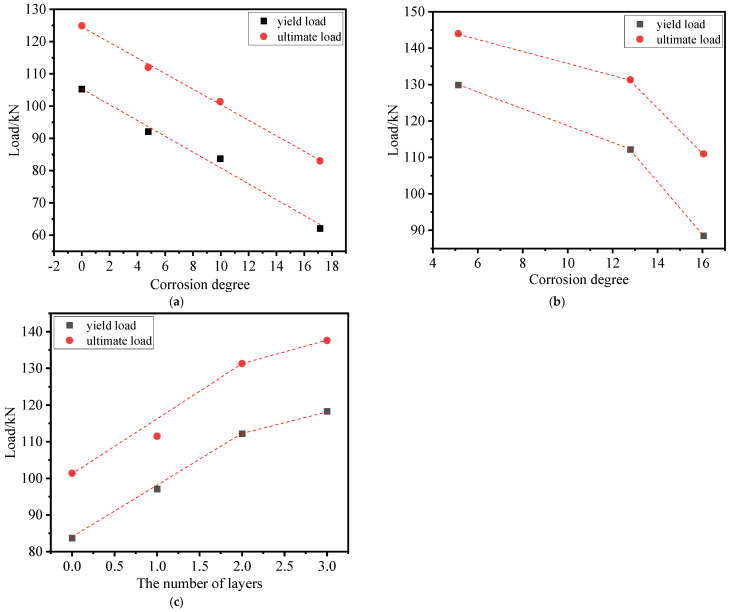
Load-different influencing factors: (**a**) M-0, M-5, M-10, and M-5; (**b**) M-5-L2, M-10-L2, and M-15-L2; (**c**) M-0, M10-L1, M-10-L2, and M-10-L3.

Table 5 presents the ultimate load and yield data for test beams. As shown in Table 5, the yield loads for test beams M-15, M-10, and M-5 experienced reductions of 41.03%, 20.51%, and 12.54%, respectively, compared to the original beam M-0. Correspondingly, the ultimate loads decreased by 10.33%, 18.82%, and 33.55%, respectively. This demonstrates the notable impact of tensile steel bar corrosion on the load-bearing capacity of RC beams, with a more pronounced reduction occurring as the corrosion degree escalates. Figure 8a illustrates the relationship among corrosion degree, ultimate load, and the yield load in corrosion-only beams, indicating that the ultimate and yield loads of the corrosion-only beam exhibited an approximately linear correlation with increasing corrosion degree. This behavior was attributed to the uniform corrosion of the steel bars induced by the electrification corrosion method.

According to the data in Table 5, specimens M-15-L2, M-10-L2, and M-5-L2 exhibited yield loads of 88.5 kN, 112.2 kN, and 129.9 kN, respectively. These values represent 143%, 134%, and 141% of the corresponding yield loads in corrosion-only beams, respectively. Furthermore, the ultimate loads for specimens M-15-L2, M-10-L2, and M-5-L2 were 111 kN, 131.3 kN, and 144 kN, respectively, reaching 134%, 129%, and 128% of the ultimate loads in the corresponding corrosion-only specimens. This observation underscores the significant enhancement in both the yield and ultimate load capacities when TRC is introduced. This improvement stems from the transfer of a portion of the tensile stress initially carried by the steel bars to the textile, compensating for the reduced load-bearing capacity owing to the steel bar cross-sectional area loss. Figure 8b depicts the relationship between the corrosion degree, ultimate load, and yield load for M-5-L2, M-10-L2, and M-15-L2. A nonlinear association existed between the corrosion degree, ultimate load, and yield load of these specimens. Specifically, the ultimate bearing capacity of specimen M-5-L2 increased by 28.57% when compared to M-5, whereas M-10-L2 and M-15-L2 exhibited increases of 29.49% and 33.74%, respectively, compared to M-10 and M-15. This suggests that the effectiveness of the TRC system in repairing beams with more severe corrosion was more pronounced.

Table 5 displays the yield loads for M-10-L1, M-10-L2, and M-10-L3 as 97.1 kN, 112.2 kN, and 118.3 kN, respectively, along with ultimate loads of 111.5 kN, 131.3 kN, and 137.6 kN. An increased number of textile layers correlated with high and ultimate loads. Figure 8c illustrates that when the amount of textile layers was less than or equal to two, the ultimate and yield loads exhibited a linear increase with the addition of textile layers. However, when the textile layer count exceeded two, a nonlinear relationship emerged between the yield load, ultimate load, and number of textile layers. This was attributed to two factors: a decrease in the utilization rate of the textile with the increase in the number of layers and the impacts of different failure modes. For instance, M-10-L3 experienced delamination failure in the strengthening layer, whereas M-10-L1 and M-10-L2 exhibited bending failures.

### 3.3. Load–Displacement Curve

**Figure 9:**
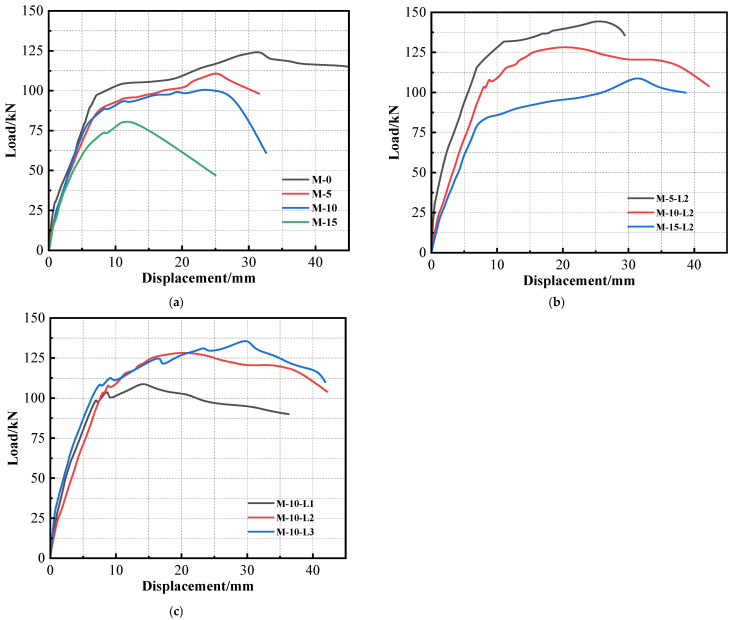
Load–deflection curves: (**a**) M-0, M-5, M-10, and M-5; (**b**) M-5-L2, M-10-L2, and M-15-L2; (**c**) M10-L1, M-10-L2, and M-10-L3.

Figure 9 presents the curves of load displacements for each specimen. Specifically, Figure 9a illustrates the curve of load displacements for the corrosion-only beam. An examination of these curves in Figure 9a reveals that, with the exception of specimen M-15, which exhibited severe corrosion with a measured rate of 17.14%, the curves for M-10, M-5, and M-0 shared a common three-stage characteristic pattern. The first stage occurred before cracking, during which the tested beam exhibited the highest stiffness. The second stage encompassed the cracking phase, which extended until the steel bars reached their yield point, resulting in a decrease in beam stiffness. The third stage encompassed the period from yielding to failure, during which the stiffness of the test beam diminished further. In stark contrast, the curve of load displacements for M-15 significantly differed from those of M-0, M-5, and M-10. M-15 did not exhibit a noticeable yield point and demonstrated minimal plastic deformation, which was indicative of brittleness. This behavior resulted from corrosion-induced deterioration of the mechanical properties of the steel bar. The research cited in [37] indicates that when the corrosion degree of the steel bar surpassed a specific threshold, its stress–strain curve underwent transformation: the yield plateau disappeared, ductility deteriorated, the softening stage vanished, and brittle fracture occurred.

Figure 9b illustrates the load–displacement curves of three specimens, M-10-L2, M-5-L2, and M-15-L2. As depicted in Figure 9b, the load–displacement curves of the TRC-strengthened corroded beams also displayed three stages: pre-cracking, cracking until steel yield, and yielding until failure. Additionally, for specimens M-15-L2, M-10-L2, and M-5-L2, the midspan displacement increased sequentially under the same applied load. The stiffness of the strengthened beam was influenced by the corrosion degree of the steel bars when the number of textile layers remained constant. A comparison between the load and displacement curves of specimens M-15-L2 and M-15 revealed that despite the degradation in that of M-15, the TRC loaded and displaced it to maintain the three-stage characteristic behavior and exhibited a certain level of ductility.

In Figure 9c, the load–displacement curves for specimens M-10-L1, M-10-L2, and M-10-L3 are presented. Examining the deflection development in relation to the actual corrosion degree of the specimens, it was observed that the pre-yielding deflection control capability was positively correlated with the number of textile layers. This correlation is attributed to the ability of the TRC layer to maintain an optimal performance with the corroded beam during this stage. A higher number of textile layers results in increased tensile stress sharing by the TRC layer, thereby retarding deflection development. As indicated in Figure 9c, it is apparent that a single layer of TRC is insufficient for effective deflection control. Notably, the deflection of M-10-L1 experienced a significant increase during this stage, while the deflection curves of M-10-L2 and M-10-L3 were closely aligned. Moreover, in the early stages of plastic deformation, the deflection of the former was smaller than that of the latter.

### 3.4. Cross-Section Verification

**Figure 10:**
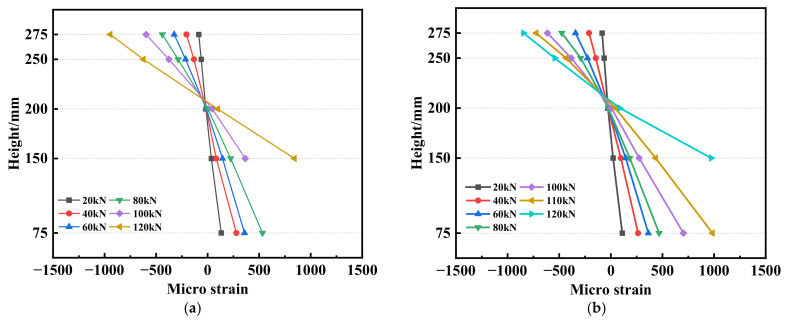
Strain distribution of concrete: (**a**) M-5-L2; (**b**) M-10-L2.

Using M-5-L2 and M-10-L2 as representative examples, we illustrated the distribution of concrete strains along the direction of the mid-span section height of the test beam. Figure 10a,b presents the correlation between the strain and load in the midspan section of M-5-L2 and M-10-L2. As shown in Figure 10, as the load continued to increase, the neutral axis of the specimens ascended, causing the strain distribution curve to shift progressively to the right. Despite this shift, the strain–distribution curve remained linear as the load increased. Consequently, it is reasonable to conclude that M-5-L2 and M-0-L2 adheres to the assumption that a flat section is maintained during the loading process. It is worth noting that the remaining test beams also conform to the plane section assumption, although they are not discussed in detail here.

## 4. Theoretical Calculation of Bending–Bearing Capacity

### 4.1. Basic Assumptions

To formulate the ultimate flexural bearing capacity model for U-type TRC-strengthened corroded RC beams, the following assumptions were made:

(1) The U-type TRC-strengthened corroded RC beams adhere to the plane section assumption during loading.

(2) Concrete in the tensile zone exits immediately after cracking.

(3) During loading, the relative slip between materials is ignored.

### 4.2. Derivation of Computational Models

**Figure 11:**
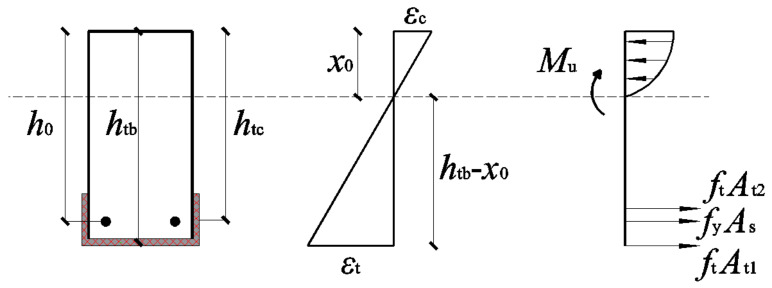
Interface stress and strain distribution map before specimen failure.

The stress–strain distribution of the cross-section of the U-type TRC-strengthened corroded RC beams before failure is shown in Figure 11.

On the verge of failure, the tensile stress in the lowermost tensile zone of the specimen was distributed among three components: the corroded steel bars, the lower TRC layer, and the side TRC layer. Upon failure, the steel bars have already yielded, the textile within the TRC layer is entirely separated, and the upper concrete of the beam is crushed.

Based on the geometric relationship depicted in Figure 11, we calculated the equivalent compression zone height, denoted as *x*_0_, using the following equation:

(3) $$\frac{\varepsilon_{t}}{h_{t}-x_{0}}=\frac{\varepsilon_{c}}{x_{0}}$$

Following the calculation of the equivalent height of the compression zone, we determined the location of the resultant force moment within the compression zone:

(4) $$M_{u}=M_{y}+M_{t1}+2M_{t2}$$

(5) $$M_{y}=f_{y,\mathrm{corr}}A_{\mathrm{corr}}(h_{0}-\frac{x_{0}}{2})$$

(6) $$M_{t1}=f_{t}A_{t1}(h_{\mathrm{tb}}-\frac{x_{0}}{2})=E_{t}\varepsilon_{t}A_{t1}(h_{\mathrm{tb}}-\frac{x_{0}}{2})$$

(7) $$M_{t2}=f_{t}A_{t2}(h_{\mathrm{tc}}-\frac{x_{0}}{2})=E_{t}\varepsilon_{t}A_{t2}(h_{\mathrm{tc}}-\frac{x_{0}}{2})$$

In this context, based on ACI 549.4R-13 [38], the maximum strain of the textile (ε_t_) and the maximum compressive strain of concrete (*ε*_c_) are 0.012 and 0.0033, respectively. The variables *h*_0_, *h*_tb_, and *h*_tc_ represent the heights measured from the point of action of the combined force exerted by the steel bar, bottom TRC layer, and side TRC layer to the top of the beam, respectively, expressed in millimeters (mm). Additionally, *x*_0_ denotes the equivalent compression zone height of the cross-section, measured in millimeters (mm). Furthermore, *A*_t1_ and *A*_t2_ correspond to the cross-sectional areas of the bottom and side textiles, respectively, in square millimeters (mm²), where *E*_t_ is the elastic modulus of the textile, which was assumed to be 246 GPa. The tensile strength (*f*_y,corr_) and effective cross-sectional area (*A*_corr_) of the corroded steel bars were obtained, using the formula provided in [39]:

(8) $$f_{y,\mathrm{corr}}=f_{y}(1-0.005\;Q_{\mathrm{corr}})$$

(9) $$A_{\mathrm{corr}}=A_{s}(1-0.01\;Q_{\mathrm{corr}})$$

where *f*_y_ and *A*_s_ represent the yield strength and cross-sectional area of the steel bar before corrosion, respectively; and *Q*_corr_ is the measured corrosion degree.

After calculating the ultimate bending moment *M*_u_, the ultimate bending bearing capacity of the TRC-strengthened beam was calculated using structural mechanics methods:

(10) $$P=\frac{2M_{u}}{a}$$

where *a* is the shear span of the specimen (700 mm).

### 4.3. Verification

This section presents the theoretical calculations regarding the ultimate bending bearing capacity of specimens M-5-L2, M-10-L1/2/3, and M-15-L2. The calculated values were then compared with the corresponding results (Table 6).

The theoretical bearing capacity calculations for all specimens aligned closely with the experimental results, exhibiting deviations predominantly within the 10% range. It is noteworthy that the theoretical bearing capacity values for all specimens were generally lower than the experimentally measured values. This disparity can be caused by the non-uniform extent of corrosion in the steel bars owing to the non-uniform nature of mass loss rates in certain areas, which may be lower than the design assumptions. However, this observation underscores the relative safety of predicting the bearing capacity of reinforced components using this formula.

## 5. Conclusions

This study conducted experiments on the flexural bearing capacity of corroded RC beams strengthened with U-type TRC. The following key conclusions can be obtained:

(1) Three distinct failure modes were identified in corroded RC beams reinforced with U-type TRC. Specifically, when the corrosion degree reached 10%, specimens with one and two layers of textile experienced bending failure, while specimens with three layers of textile exhibited TRC lateral peel failure.

(2) The TRC strengthening system proved to be highly effective in enhancing the flexural bearing capacity of the corroded RC beams. Notably, both the ultimate and yield loads increased with the increased textile layer count.

(3) In the case of mild and moderate corrosion levels in beams, employing TRC with more than one layer of textile proved capable of not only restoring but also surpassing the original bearing capacity. However, for beams exhibiting severe corrosion, the use of two textile layers is insufficient to restore their bearing capacity to the level of the original beam.

(4) A theoretical equation for determining the ultimate bearing capacity of corroded RC beams strengthened with U-type TRC was developed, and it demonstrated a high level of accuracy in its calculations.

## Figures and Tables

**Table 1:** Mechanical properties of concrete.

Spec.	Compressive Strength/Mpa	Avg/Mpa	Cv
1	37.20	38.40	0.022
2	38.40		
3	39.30		

**Table 2:** Mechanical properties of steel bar.

Spec.	Yield Strength/Mpa	Ultimate Strength/Mpa	Elastic Modulus/Gpa	Elongation/%
1	438	616	192	23.45
2	442	617	188	21.96
3	440	609	194	22.42
Avg	440	614	191	22.61
Cv	0.004	0.006	0.013	0.028

**Table 3:** Mechanical properties of carbon textile [32].

Direction of Fiber	Tensile Strength/MPa	Ultimate Strain/%	Elastic Modulus/GPa
Longitudinal	5110	2.1	246
Transverse	4815	1.9	252

**Table 4:** Mechanical properties of matrix.

Spec.	Compressive Strength/Mpa	Avg/Mpa	Cv
1	42.60	42.97	0.006
2	43.10		
3	43.20		

**Table 5:** Test results’ summary.

Spec.	*η/%*	P_y_ (kN)	δ_y_ (mm)	P_u_ (kN)	δ_u_ (mm)	(P_y_ − P_y,M-0_)/P_y,M-0_	(P_u_ − P_u,M-0_)/P_u,M-0_
M-0	-	105.3	10.34	124.9	44.76	-	-
M-5	4.78	92.1	9.47	112	31.55	−12.54%	−10.33%
M-10	9.95	83.7	9.36	101.4	29.4	−20.51%	−18.82%
M-15	17.14	62.1	6.67	83	17.37	−41.03%	−33.55%
M-5-L2	5.13	129.9	8.33	144	29.42	23.36%	15.29%
M-10-L2	12.79	112.2	10.27	131.3	39.44	6.55%	5.12%
M-10-L1	11.01	97.1	8.79	111.5	32.02	−7.79%	−10.73%
M-10-L3	12.42	118.3	9.45	137.6	40.91	12.35%	10.17%
M-15-L2	16.05	88.5	12.16	111	41.83	−15.95%	−11.13%

Notes: P_y_ = yielding load, δ_y_ = mid-span deflection at the yielding load, P_u_ = ultimate load, δ_u_ = mid-span deflection at the ultimate load, P_y,M-0_ = yield load of M-0, and P_u,M-0_ = ultimate load of M-0.

**Table 6:** Comparison between theoretical calculation values and experimental values.

Spec.	P_u_ (kN)	P_th_ (kN)	P_u_/P_th_
M-5-L2	144.0	136.20	1.06
M-10-L1	111.5	110.18	1.01
M-10-L2	131.5	122.67	1.07
M-10-L3	137.0	133.40	1.03
M-15-L2	111.0	102.21	1.09

Notes: P_u_ = actual measured ultimate load, P_th_ = calculated ultimate load.

## Data Availability

Data are contained within the article.

## Nomenclature

*x*_0_	the equivalent compression zone height
ε_t_	the maximum strain of the textile
*ε*_c_	the maximum compressive strain of concrete
*h*_0_	the heights measured from the point of action of the combined force exerted by the steel bar to the top of the beam
*h*_tb_	the heights measured from the point of action of the combined force exerted by the bottom TRC layer to the top of the beam
*h*_tc_	the heights measured from the point of action of the combined force exerted by the side TRC layer to the top of the beam
*A*_t1_	the cross-sectional areas of the bottom textiles
*A*_t2_	the cross-sectional areas of the side textiles
*E*_t_	the elastic modulus of the textile
*f*_y_	the yield strength of the steel bar before corrosion
*A*_s_	the cross-sectional area of the steel bar before corrosion
*f*_y,corr_	the tensile strength of the corroded steel bars
*A*_corr_	the effective cross-sectional area of the corroded steel bars
*Q*_corr_	the measured corrosion degree
*M*_u_	the ultimate bending moment
*a*	the shear span of the specimen
